# Survey on Prominent RFID Authentication Protocols for Passive Tags

**DOI:** 10.3390/s18103584

**Published:** 2018-10-22

**Authors:** Rania Baashirah, Abdelshakour Abuzneid

**Affiliations:** Department of Computer Science and Engineering, University of Bridgeport, Bridgeport, CT 06604, USA

**Keywords:** RFID, security, privacy, authentication, passive tag, security threats, security attacks, IoT, lightweight protocol

## Abstract

Radio Frequency Identification (RFID) is one of the leading technologies in the Internet of Things (IoT) to create an efficient and reliable system to securely identify objects in many environments such as business, health, and manufacturing areas. Recent RFID authentication protocols have been proposed to satisfy the security features of RFID communication. In this article, we identify and review some of the most recent and enhanced authentication protocols that mainly focus on the authentication between a reader and a tag. However, the scope of this survey includes only passive tags protocols, due to the large scale of the RFID framework. We examined some of the recent RFID protocols in term of security requirements, computation, and attack resistance. We conclude that only five protocols resist all of the major attacks, while only one protocol satisfies all of the security requirements of the RFID system.

## 1. Introduction

The wireless sensor network has expanded recently to employ new technologies in the Internet of Things (IoT). The purpose of this evolution is to create a low-cost, reliable, and secure communication network for current and future applications using radio waves in the most convenient way. Radio Frequency Identification (RFID) is a technology where the detection of the electromagnetic signals in the wireless sensor network identifies objects or people. Hundreds and thousands of RFID applications have been used to improve business efficiency and productivity in a variety of business operations, including supply chain management, access control limitation, product tracking, merchandise allocation, toll collection, and so on. It is also considered an integral part of daily life where its applications not only are limited to business activities, but also daily life activities that are integrated into cell phones, household, automobile, etc.

Although the basic concept of RFID is similar to barcodes in identifying the items using the data stored in barcodes, RFID technology has vital benefits over barcodes. It does not require physical contact with the objects, allows scanning multiple and different types of barcodes using one signal, has the ability to read and write on the tag multiple times [1], and enables identifying objects in different climates such as fog and snow, and packaging conditions such as ice, perishable food, and liquids [2].

RFID is considered a significant structure for future market development. Many business enterprises and manufactures nowadays in the supply chain, including banks, transportation, government, agriculture, food safety, health care, and mass production, are using RFID to automate their product identification faster in different conditions to improve their business efficiency and customer service experience.

## 2. System Architecture and Communication Model

**Figure 1:**
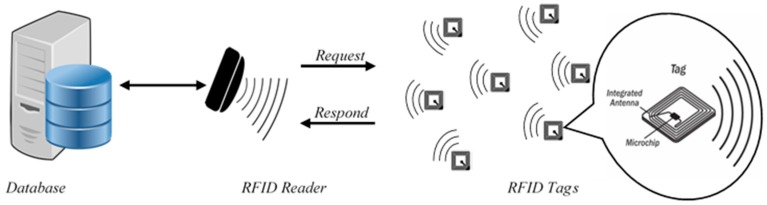
Basic Radio Frequency Identification (RFID) Model.

The basic system of RFID includes a receiver (reader), transponder (tag), and back-end database (server) to store and manage data. The RFID tag is a label that is placed into the object to be identified and located among hundreds and thousands of objects. It consists of a small antenna attached to a microchip with a small memory to store the object’s identity and data [3]. The RFID reader is a scanner placed in a fixed location to interrogate the tag whenever the tag exists in the scanning environment. The back-end database server operates as a data processor that manages, controls, and stores the data from the tag and reader. An RFID system is depicted in Figure 1 [4].

RFID tags can be classified into three categories based on the storage memory, cost, and battery requirements: passive tags, semi-passive tags, and active tags [5,6].

- A passive tag operates without battery, as the tag is energized when the reader interrogates it by sending a signal to request tag information. It has a short transmission range in communication, and has limited resources in term of storage. It is considered the lowest in cost and has a higher lifespan.
- A semi-passive tag has a battery for its internal chip circuit; however, it is also energized by the reader interrogation, as in the passive tag.
- An active tag runs with battery and can have two-way communication between tag and reader. It is larger due to the larger storage capacity and battery. The transmission range is also larger compared to passive tags. It is more expensive and has a limited life depending on the battery lifespan [2].

Table 1 provides some comparison of the three types of RFID tags.

The basic communication session between an RFID reader and a tag starts when the reader broadcasts radio waves to interrogate the tag. The tag receives the signal and responds corresponding to the reader’s request. Since the communication channel between the reader and tag is assumed to be insecure, it is important to maintain a secure system during communication to avoid information leakage or forgery by unauthorized users. Efficient RFID concerns about system security, cost, and liability are essential factors for future adoption in the IoT.

## 3. Security Requirements and Threats

### 3.1. Security Requirements

The basic entities in the RFID system are the tag, reader, and database server. The communication channel between a tag and a reader is insecure and vulnerable to different security threats. Security requirements are the ability features that enable the system to avoid security threats. There are several security requirements to evaluate the security level of an RFID system:

- *Mutual Authentication*: the main requirement in a simple scenario of RFID communication session is the authentication between the reader and tag before exchanging or transmitting any secret or valuable information. Both tag and reader have to prove their legitimacy to each other to start a secure communication.
- *Confidentiality*: all of the transmitted messages have to be secure in which secret information and values that are used to execute communication cannot be obtained by an unauthorized user.
- *Integrity*: the transmitted data has to maintain its accuracy and not to be altered or changed during communication.
- *Availability*: the communication should be successfully executed by maintaining a synchronous state between the RFID entities. Communication values have to be updated after every successful session to provide system availability.
- *Privacy*: all of the secret information such as tag identity has to be secured in order to provide anonymity and avoid tracing the tag or its location.
- *Forward Security*: the transmitted data during communication have to be independent and updated for every session, and cannot be used or related to another authentication session. If a tag or any information is compromised, it is impossible for an adversary to pass the authentication on or violate the system.

### 3.2. Security Threats

A secure RFID system must be able to resist different types of attacks. Messages in RFID communication are transmitted in clear, and thus are vulnerable to eavesdrop; hence, secret information is disclosed. Many RFID protocols are proposed to defend against different attacks such as:

- *Replay Attack*: an adversary tries to capture the tag response and resend it to the reader to start a successful communication with the reader or obtain any secret information.
- *Man-In-The-Middle*: an adversary intercepts the message between two legitimate entities tag/reader to modify it and send it back.
- *Impersonate Attack*: an adversary obtains either the reader or tag identity information to create a forged entity. As a result, the adversary acts as a legitimate entity to pass the authentication and proceed with the communication.
- *Traceability*: an adversary traces the tag to find its location and revoke the tag’s privacy. This attack violates the private information of RFID users, which is an instance where the privacy is important.
- *Desynchronization Attack*: communication session between tag and reader starts using the synchronous values stored in both the tag and reader to authenticate each other. A desynchronization attack occurs when an adversary breaks the synchronous state between the tag and server by blocking the update messages, causing the communication values stored in both server and tag to be different.
- *Denial of Service*: an adversary sends multiple signals simultaneously to the server as responses to make the system unavailable for further communication, which could further lead to a desynchronization attack.
- *Cloning*: an adversary uses a malicious device to obtain the reader or tag secret information and create a fake entity that can be used to perform a successful communication.
- *Disclosure*: an adversary identifies the secret information of the tag and the secret keys used in the communication to fully compromise the security of the protocol.

Many other security threats have been identified for RFID systems. A secure RFID system is created to defend against various threats that are related to the application in use.

## 4. Review of Recent RFID Authentication Protocols

Several articles are proposed to create a secure RFID protocol that improves the security measures of RFID systems. The modern advancement in technology helps discover many gaps in the proposed protocols presented in the literature. The aim of this work is to review some of the recent RFID authentication protocols that specifically use passive tags. We aim to present an adequate comparison between the protocols in terms of performance and security. 

Since a passive tag is a very small chip with scarce resources, it is able to do only low computations. Hence, RFID protocols are classified in this paper into four categories based on the *complexity of the algorithm* that is used to compute the tag responses: heavyweight, simple weight, lightweight, and ultra-lightweight [9]. Heavyweight algorithms use symmetric and public key cryptography that is beyond the scale of the passive tag ability to process. Simple-weight algorithms use hash functions that are also not feasible for passive tag resources. Lightweight algorithms use simple one-way hash functions, cyclic redundancy checks, and pseudo-random number generators [10]. Finally, ultra-lightweight algorithms use bitwise operations, which can be performed at low cost.

### 4.1. Heavyweight Protocols

**Figure 2:**
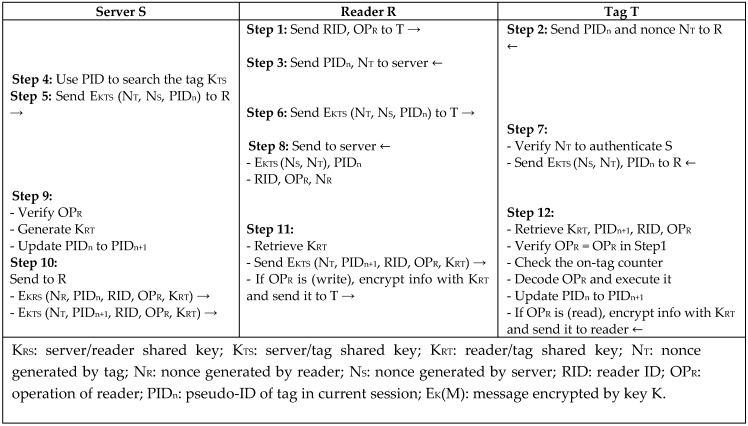
Session-Based Authentication Protocol (SB-A) by Wang and Sarma.

**Figure 3:**
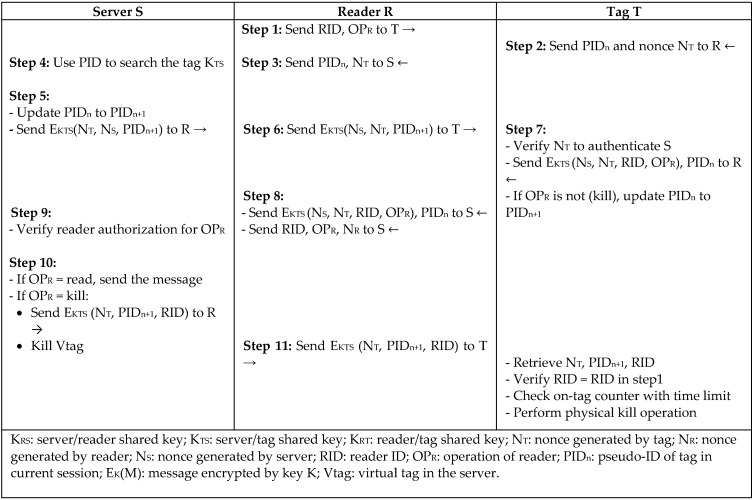
Session-Based Authentication Protocol (SB-B) by Wang and Sarma.

Wang and Sarma [11] proposed two session-based authentication protocols, *SB-A* and *SB-B*, for reader–tag authentication based on symmetric key encryption to ensure privacy and access control using two types of passive tags. The protocols are based on a symmetric cryptography algorithm to provide low-cost authentication such as the Advanced Encryption Standard (AES) and Data Encryption Standard (DES). Protocol *SB-A* in Figure 2 includes two processes. The first phase involves mutual authentication between server and tag according to the three-pass mutual authentication protocol according to the International Organization of Standardization and the International Electrotechnical Commission—ISO/IEC 9798-2 [12]. The second phase is for generating a session key between reader and tag according to the Otway–Rees protocol and updating the pseudo tag identity (PID). Protocol *SB-B* in Figure 3 uses tags with no memory or ID so that all of the tag’s information is stored in the server. A physical tag operation is mapped with the digital virtual tag in the server that can do all of the tag’s executions. The protocol time to keep synchronization is controlled by the tag nonce and counter, and not the server, because of the limited power of the tag to keep synchronization. The protocols proved to be secure against major types of attacks; however, the protocols are considered to be heavyweight, since DES and AES are expensive operations that require a lot of computational overhead. 

**Figure 4:**
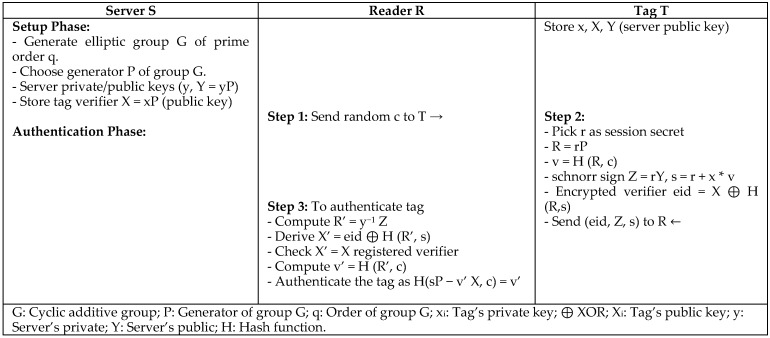
Elliptic Curve Cryptography-Based Untraceable Authentication Protocol (ECU) by Ryu.

For traceability issues in RFID, Ryu et al. [13] proposed elliptic curve cryptography-based untraceable authentication protocol (ECU) using the Schnorr signature scheme. The elliptic curve cryptography is considered to be a public key cryptography for RFID systems with low constrained tags. It is used to solve the issues of three recent elliptic curve-based untraceable RFID authentication protocols: Strong Privacy-preserving Authentication protocol (SPA) [14], Efficient Mutual Authentication protocol EMA [15], and ECC-based authentication protocol PII [16]. Ryu’s protocol generates a digital signature with an appendix on the binary message of arbitrary length, and requires a cryptographic hash function, as shown in Figure 4. The sender’s session key is combined with the receiver’s public key to provide privacy, in which the message can be verified by only the receiver’s private key. Ryu’s protocol is secure against replay attacks, impersonate attacks, traceability attacks, and it maintains forward security. It requires two scalar multiplications, two hash functions, a message total size of 544 bits, and two communications between tag and reader. Even though this protocol requires complex computations associated with scalar multiplications and a hash function, it does not authenticate the reader. 

**Figure 5:**
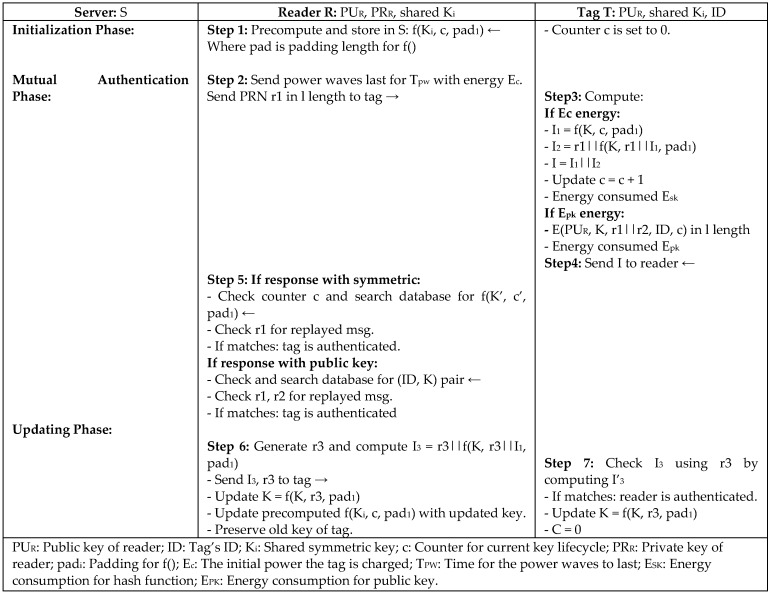
The Reviving-UNder-Denial of Service Authentication Protocol (RUND) by Yao.

To reduce the tag’s overhead in heavyweight protocols, Yao et al. [17] introduced The Reviving-UNder-DoS (RUND) authentication protocol to defend against denial of service (DoS) and preserve user privacy by powering up the tag to do complex computing for symmetric and public key cryptography. It leverages the power in DoS scans to enable the tag to respond in two ways: either using simple encryption when the tag is activated by low signals from a reader, or using public encryption (higher security) when the backscattered signals are high in an insecure environment. The more signals there are in communication, the more power charges the tag. The option of using public key encryption in RUND protocol is to overcome the problem of breaking up the synchronization state between the reader and tag in symmetric key encryption. The protocol is secure because secret information is not sent in clear, so no useful information can be gained if any message is compromised. Moreover, the parameters used in communication are changed and updated in every session, as shown in Figure 5, to prevent replay attack, maintain forward security, and resist tracking. Even though the overall efficiency of RUND is O(1), it is still not compliant with the Electronic Product Code Class1 Generation2 (EPC C1 G2) standard [18], which is defined by EPCGlobal Inc. for RFID data communication. 

### 4.2. Simple-Weight Protocols

**Figure 6:**
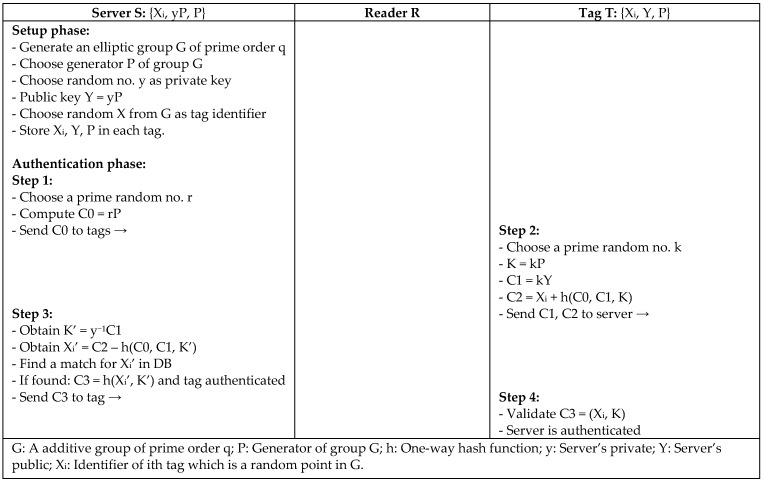
Mutual Authentication Protocol Based on Elliptic Curve Cryptography (IECC) by Farash.

To better improve the performance of RFID protocols and reduce the power that is needed for complex operations in ECC-based protocols, Farash [19] proposed a mutual authentication protocol (IECC) based on the elliptic curve. The protocol enhances Chou’s authentication protocol (EMA) [15], which does not fulfill the security requirement of forward security, mutual authentication, tag privacy, and security against location tracking, impersonating attacks, and tag cloning attack for an RFID system. The main idea behind the protocol is to use the server’s public key to create the authentication message to avoid breaking the system privacy, as depicted in Figure 6. The IECC protocol is secure against major attacks, even though the computation cost is the same as in Chou’s protocol that needs to be reduced for practical implementation.

Zhang and Qi [20] also proposed another protocol (EECC) to withstand the security weaknesses of Chou’s protocol, EMA [15]. EECC protocol enhances patient medication safety by also using elliptic curve cryptography. In comparison to EMA protocol, EECC protocol resulted in better performance and security resistance to impersonate and forward security attacks.

**Figure 7:**
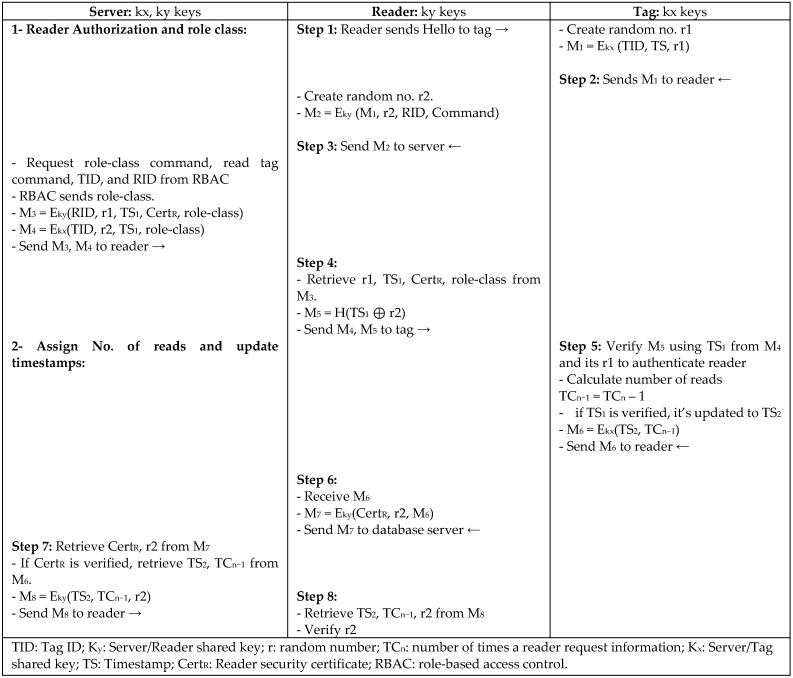
Role-Based Access Control Protocol (RBAC) by B. Chen.

B.Chen [21] proposed a role-based access control (RBAC) protocol for mobile RFID to enable user privacy, role, and access control through the back-end server based on a certification mechanism. RBAC assigns role classes as keys to control the information and the number of times each reader can read a tag. RBAC authorizes readers, assigns role classes to control the reader’s authority to request tag information, and updates time stamps using random numbers and different shared keys between the database server and reader and tag ad, as depicted in Figure 7. Traceability and replay attacks are prevented using updated random numbers in every session; access control is provided using shared keys to prevent unauthorized readers to request or read any tag’s information, and integrity is ensured using timestamps. However, RBAC uses one encryption mechanism that is excessive for low-cost passive tags.

### 4.3. Lightweight Protocols

Successful businesses demand an efficient RFID system that is mainly based on low computation for a low cost. Many recent RFID protocols use low-cost operations that are handled by low-cost passive tags for practical implementations.

**Figure 8:**
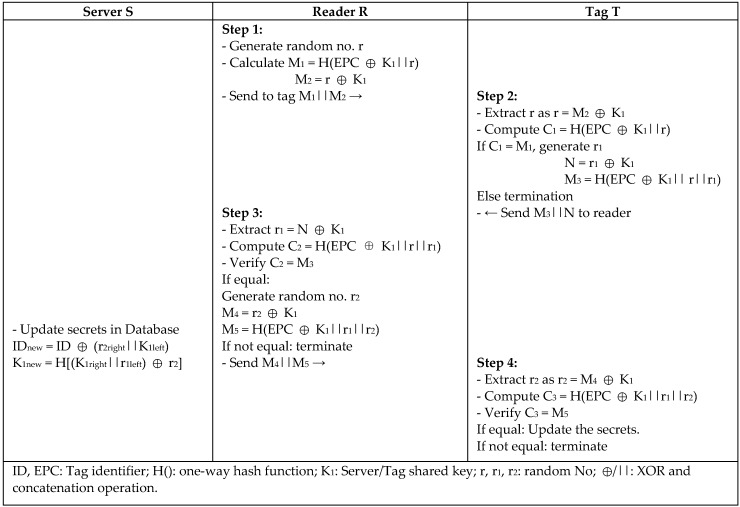
Mutual Authentication Protocol for Networked RFID Systems (NRS++) by X. Chen.

Fernando and Abawajy [22] proposed a mutual authentication protocol for Networked RFID Systems NRS, which is a lightweight mutual authentication scheme for an RFID system using low operations such as excusive or operation (XOR) and one-way hash functions. However, Alagheband and Aref [10] reported NRS to be vulnerable to major attacks and specifically a full disclosure attack that compromises the whole RFID system. Alagheband and Aref improved NRS protocol and proposed NRS+ by adding three more hash functions to the authentication message to increase the system security. X. Chen et al. [23] noted that the NRS+ protocol is exposed to desynchronization and traceability attacks by using one random number for the tag and reader. Thus, X. Chen proposed NRS++ to improve the security flaws in the previous versions of NRS by generating two different random numbers, *r1* and *r2*, for the tag and reader using a pseudo-random number generator (PRNG) to defend against replay attack. In Figure 8, the authentication message *M3* is encrypted using the tag’s random number *r1* and reader’s random number *r2* to provide message integrity, so any modified message cannot be verified by the tag. NRS++ uses fewer hash functions, which resulted in less computation overhead and storage space than the other versions, with more security power. 

**Figure 9:**
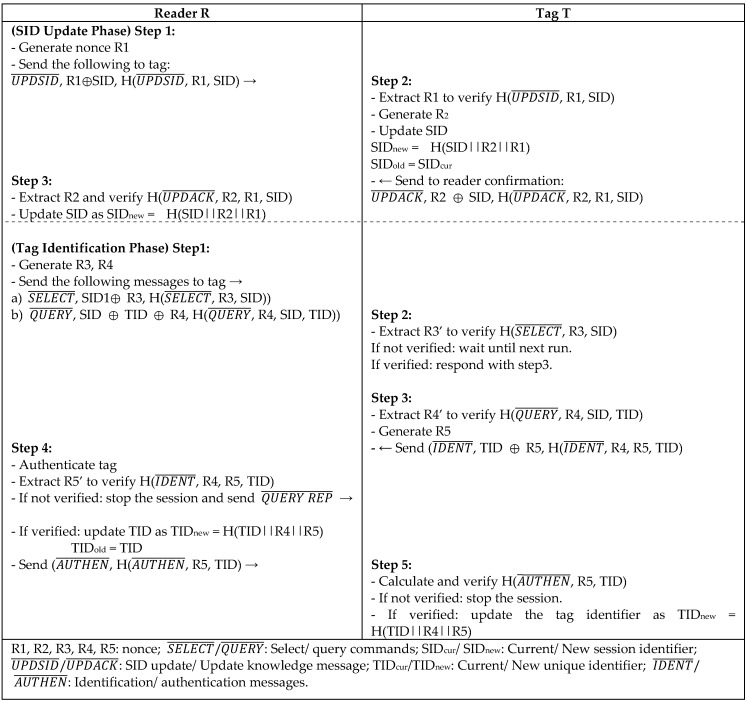
Anti-Counting Security Protocol (ACSP++) by X. Chen.

C. Chen [24] proposed Anti-Counting Security Protocol (ACSP) as another lightweight protocol for RFID systems to defend from a counter attack, which is defined as the attacker’s ability to count the number of objects in a system. Safkhani et al. [25] reported ACSP to be vulnerable to major attacks, including the forward/backward traceability attack. Safkhani further proposed ACSP+ to improve Chen’s protocol. Later, X. Chen [23] pointed out that ACSP protocol is not secure, and proposed ACSP++ to withstand DoS and forward/backward traceability attacks. ACSP++ enhances the session identifier (SID) update, which is used to verify the current session, and tag identification phases that suffer from different attacks in ACSP and ACSP+ versions. In ACSP++ as depicted in Figure 9, a tag identifier (TID) is added to the identification message as ($\bar{IDENT}$, R4, R5, TID) instead of ($\bar{IDENT}$, R4, R5), and the authentication message ($\bar{AUTHEN}$, R4, R5, TID) is replaced with ($\bar{AUTHEN}$, R5, TID) to overcome DoS attack and modifying the TID in the identification phase. The update phase of every key is associated with two separate nonce values to avoid forward and backward traceability. Even though the protocol improved the security weaknesses of all of the ACSP versions, it did not lower the computation overhead nor the storage space.

**Figure 10:**
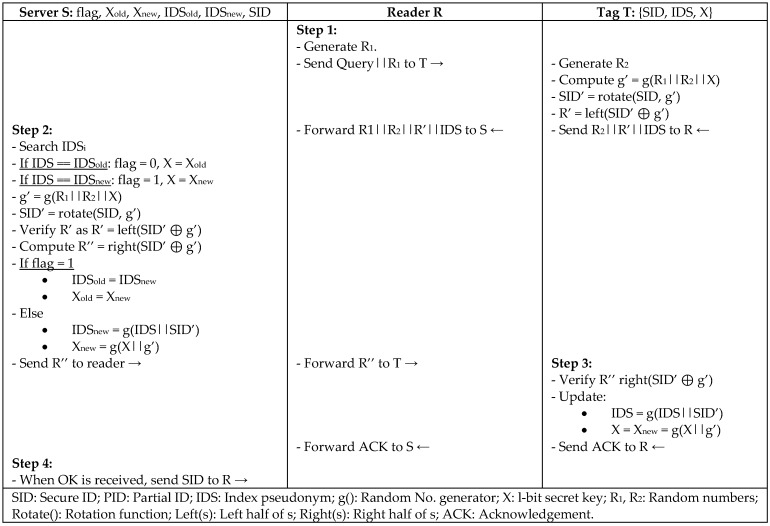
Lightweight Authentication Protocol (LAP) by Chien.

Chien and Huang [26] presented LAP, which is a lightweight authentication protocol to solve the vulnerabilities in the authentication protocol of Li et al. [27], and enhance the computational cost from O(n) to O(1) in identifying tags in RFID systems. The security of LAP protocol is based on a synchronized PRNG between reader and tag using a secret key, secret ID, and index pseudonym. In Figure 10, LAP protocol uses the rotate operator on the message and left/right operator for the divided rotation during the messages that were exchanged to form a secure permutation. Random numbers are used to shift the secret values of the tag to be used safely in communication. Then, the random number is XORed with the shifted secret value to securely retrieve a tag by the server. The server uses the index pseudonym (IDS) to quickly identify the tag in the database instead of computing PIDL ⊕ PIDR for every tag to make the computation O(1). LAP protocol is resistant to replay attack, DoS, and forward security. It can be employed easily by different standards such as EPC Gen2 and ISO 15693 [28] for practical implementation. However, the protocol was noted as being partially secure against traceability and synchronization attacks, since a tag can be traced between two successful sessions if the tag could not update its IDS. 

**Figure 11:**
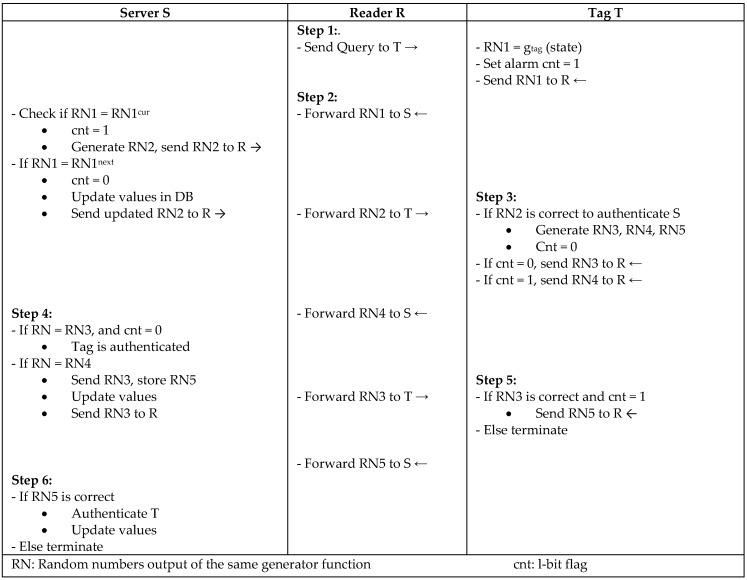
Flyweight Mutual Authentication Protocol by Burmeter and Munilla.

Burmester and Munilla [29] proposed a lightweight mutual authentication protocol called Flyweight that is based on exchanging messages using only PRNG. Their protocol is based on a shared PRNG algorithm between the tags and back-end server that takes the same seed to produce the same output. The concept of the protocol is to use three consecutive numbers—RN1, RN2, and RN3—generated by the same PRNG in the server, and the tags of five numbers if an active adversary is presented, such as in Figure 11. Furthermore, RFID tags precompute the values to the server challenging the response, so an adversary can be detected based on the response time from the tag. The protocol is able to provide mutual authentication, integrity, confidentiality, and forward and backward security. In addition, it provides strong synchronization, since the server keeps a record for the current and next response value of the tag.

**Figure 12:**
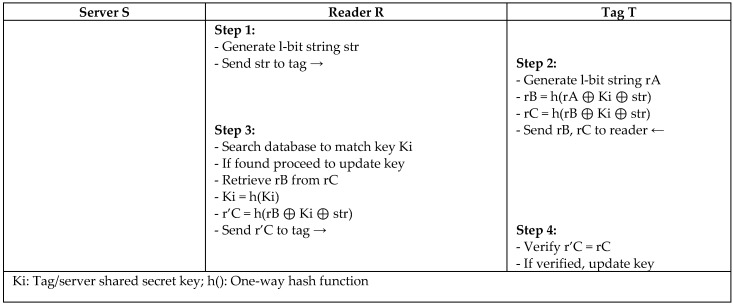
Lightweight Protocol based on Synchronized Secret (MASS) by S. Lee.

S. Lee et al. [30] proposed a lightweight protocol (MASS) for RFID systems using XOR and a one-way hash function to conform to the scarce resources of RFID tags. The concept of the MASS protocol is to challenge the tag with a fresh random string every session, and the tag responds using the reader’s value and its own random key to authenticate the reader ad, as depicted in Figure 12. The secret key is shared between entities, and all of the messages are encrypted during transmission. However, Zuo [31] conducted a survivability experiment on the authentication protocol proposed by S. Lee et al. and defined the vulnerability of the protocol to replay, desynchronize, and impersonate attacks. Zuo concluded from his experiment that the system could employ two different values for the keys (old, new) to recognize the tag and overcome the desynchronization problem. 

**Figure 13:**
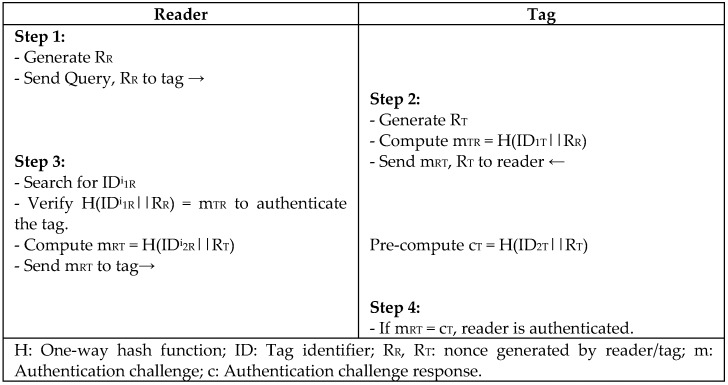
Efficient Passively-Untraceable Authentication Protocol (EP-UAP) by K. Lee.

To reduce the communication time during the authentication session, K. Lee et al. [32] proposed Efficient Passively-Untraceable Authentication Protocol (EP-UAP). The concept of EP-UAP is that the system precomputes all of the necessary computations before the system initialization, so only low computation overhead is required on the tag side during the process phase. The protocol is based on Randomized Hash-Lock protocol, which uses a static identifier, and its strong security against traceability depends mainly on PRNG to randomize the responses, as explained in Figure 13. Since precomputing all of the possible random numbers and responses requires a storage memory for all of the precomputed data in the database, EP-UAP is preferred for small to medium networks, as the storage memory increases when the number of tags increases. The protocol shows a huge improvement over the randomized hash lock protocol in terms of computation time, in that only requires 40 ms for authentication; this is similar to LRMAP, which is the most efficient one in stateful protocols. However, it requires 100 MB of database storage memory. The protocol provides integrity due to the two randomly generated nonce values that are used from both tag and reader, and is secure against passive attacks and traceability due to the random responses. However, the EP-UAP protocol seems to be vulnerable to active attacks such as impersonate and replay attacks, since the random responses depend on the database/reader. It also requires high storage capacity in the database side. 

**Figure 14:**
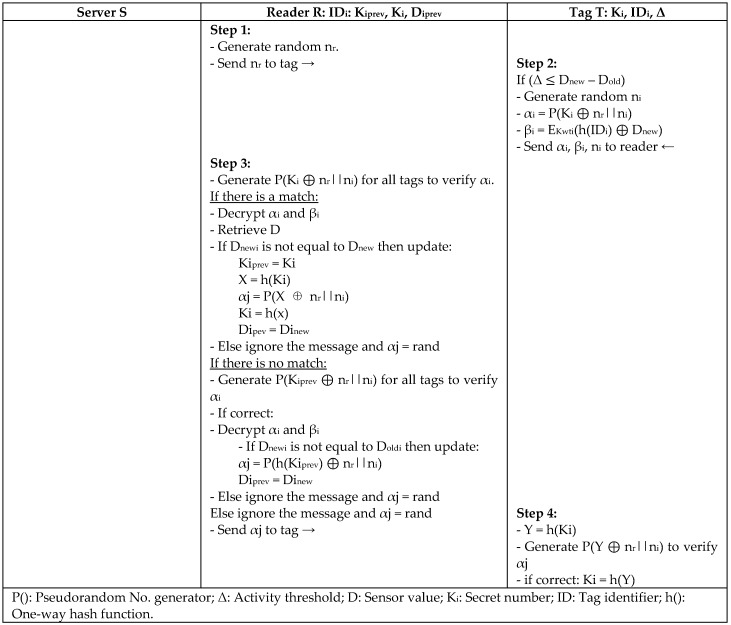
Desynchronization Attack-Resistant Robust Authentication Protocol (DRAP) by Rahman and Ahamad.

To defend against a desynchronization attack, Rahman and Ahamad [33] proposed a Desynchronization attack-resistant Robust Authentication Protocol (DRAP) in the wireless identification and sensing platforms (WISP), where RFID technology is combined with sensor nodes. Their protocol mechanism is to decrease the tag collision that leads to DoS attack, as shown in Figure 14. The technique is to decrease the collision rate at the link layer and maintain the system’s efficiency. The protocol also detects the DoS attack and recovers the synchronization state of the system. It has higher resources than passive tags, which allow higher security implementation. Yet, it has a short distance limitation, where tags can only function less than 1–2 m away from readers. 

**Figure 15:**
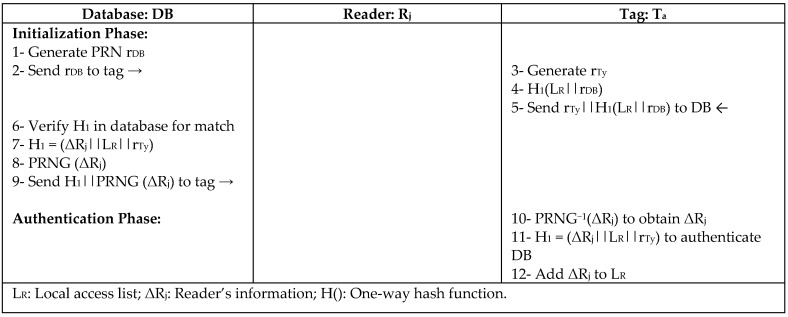
Grouping Proofs-Based Authentication Protocol (GUPA) by Liu for a Single-Reader—Single-Tag Case.

Authentication in most RFID protocols is executed between one reader and one tag at a time. Liu et al. [34] proposed a grouping proofs-based authentication protocol (GUPA) to enable authenticating multiple tags and multiple readers simultaneously, such that multiple readers can authenticate a single tag, and multiple tags can be authenticated by a single reader in large-scale RFID. GUPA protocol is based on hierarchical identification between independent subgroups in a distributed RFID system, and the use of an asymmetric denial mechanism to resist denial-of-proof attack (DoP). For the anonymous authentication of a new entity, GUPA deploys a ring signature using a lightweight cryptography (elliptic curve). It also uses lightweight bitwise operations for readers and tags secret information updates, PRNGs, one-way hash functions, timestamps for session freshness, and access lists for each legal reader/tag during system initialization as identity flags to prevent forgery and tracking attack, as fully explained in Figure 15. Since the flags are chosen randomly from the pseudonym index, queries and responses are independent for each session to resist DoP attack; hence, illegal proofs are eliminated during authentication. 

**Figure 16:**
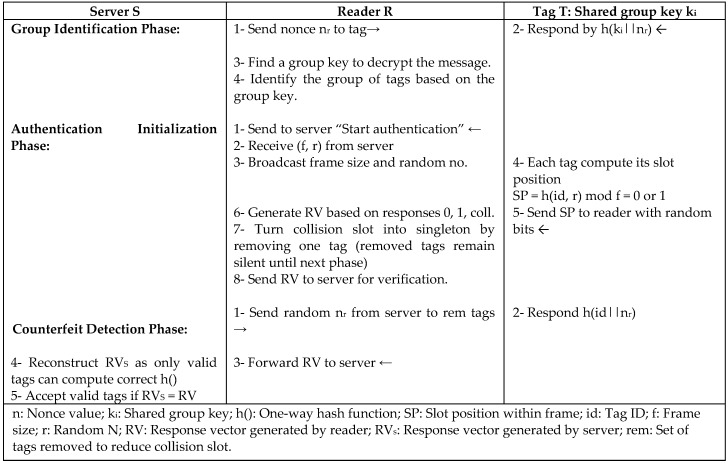
Batch Authentication Protocol based on Frame Slotted Aloha (FTest) by Rahman and Ahamad.

Since tag collision is a major problem in the large-scale networks, Rahman and Ahamad [35] proposed two probabilistic batch authentication protocols to determine the valid tags efficiently and accurately in large-scale systems. FTest is a protocol based on Frame Slotted Aloha algorithm that is used to reduce the probability of collision slots. The other protocol is GTest, which is a protocol based on group batch authentication that is used to reduce the cost of detecting counterfeit tags. Their protocols use simple lightweight operations such as XOR and cyclic redundancy checks (CRC) with a shared key for each group of tags. The theory in both protocols is not to send the tag ID when responding, but rather accept or reject a tag by estimating the number of fake tags. In the FTest protocol that is depicted in Figure 16, a counterfeit threshold parameter is used in the system to reduce the number of rounds in the detection process and response time of the protocol, so that the entire tag responses do not need to be checked. Instead, the detection will stop if the percentage of counterfeit tags exceeds the counterfeit threshold. In GTest, the reader randomly selects a population of tags to authenticate. If one counterfeit tag is detected, the batch of tags will be considered invalid. The reader needs to read a large amount of data to identify the validity of a batch in GTest, so the reader still consumes time through the computation overhead from the tag search. Both FTest and GTest protocols are proved to be secure against tracking and privacy attacks, since tags responses are based on dynamic frame size, random numbers, and ID that is not transmitted during communication. However, the FTest shows less execution time and better performance over GTest. 

**Figure 17:**
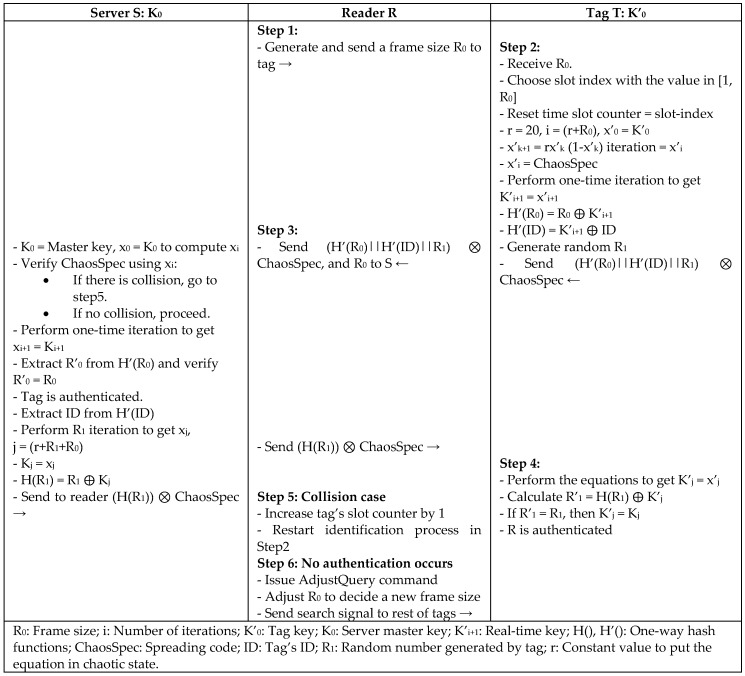
Anti-Collision Security Protocol (ACS) by Keqiang.

Another anti-collision security protocol (ACS) is proposed by Keqiang et al. [36] for a high-efficiency RFID system combining the chaotic sequence generator with the dynamic frame-slotted ALOHA algorithm for fast tag identification. The protocol scheme is based on a logistic mapping structure with XOR operation and spreading operation to generate real-time keys in a chaotic sequence that are used in authentication messages. Keys are updated in each response from tag to reader and reader to tag during the same session using iteration equations that are known only to the server and tag, such as in Figure 17. The protocol is effective against counterfeits and impersonates attacks, as the authentication scheme not only depends on the iterated key, but also on spreading code and random numbers, so faking at least one of them will result in a wrong response. The protocol requires only four message exchanges, low hardware cost, and low computation cost on the tag side. It also has lower energy consumption than other heavy and simple weight protocols, because XOR uses less energy than symmetric encryption and hash functions. 

**Figure 18:**
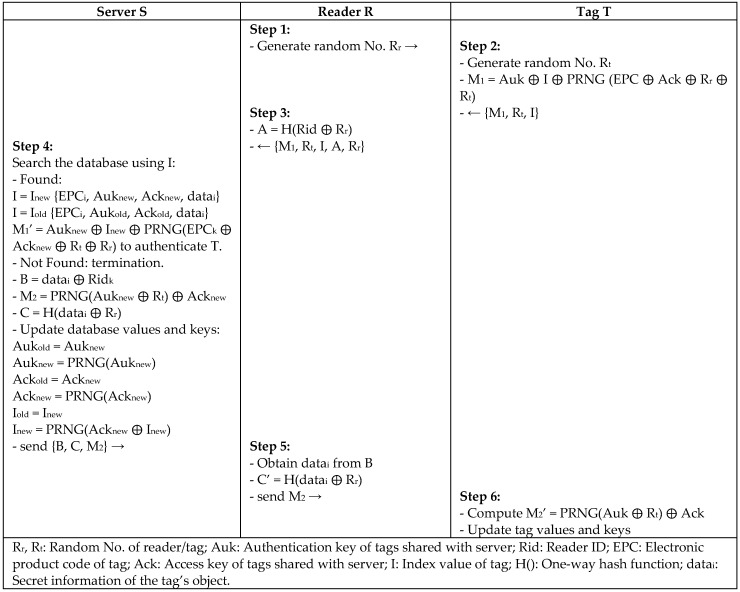
Hash-Based Mutual Authentication (HBA+) Protocol by Chang.

Cho et al. [37] proposed a hash-based mutual authentication protocol (HBA) to defend against the brute force attack. This protocol was reported by Chang et al. [6] to be vulnerable to denial of service (DoS) and replay attacks. Later, Chang et al. proposed an improved (HBA+) protocol to avoid DoS and replay attacks using a shared PRNG algorithm between the server and tag to produce the same output that is used in updating the protocol values, as in Figure 18. Also, the confidentiality in the protocol is based on protecting the secret value *datai* using reader ID (*Rid*), which is only known to a legitimate reader and server. The improved protocol of Chang is considered to be efficient and secure against DoS attack, traceability, and forward secrecy. 

**Figure 19:**
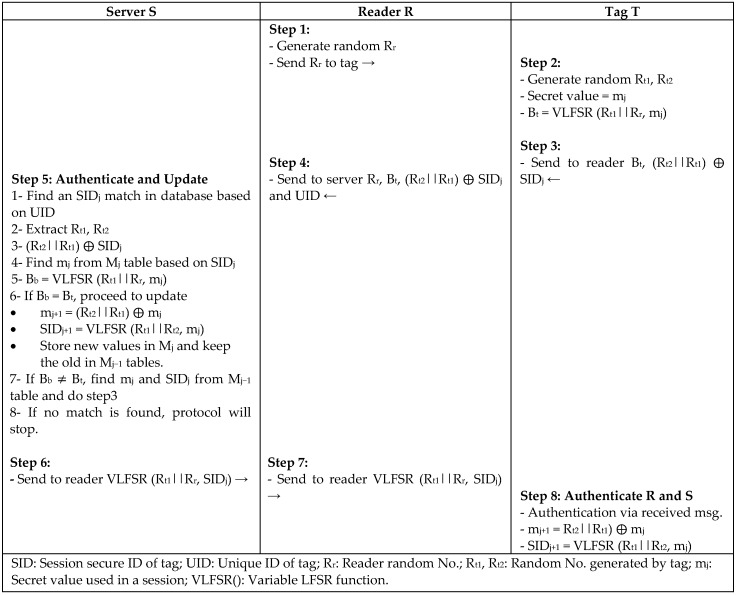
Variable Linear Shift-Based Authentication Protocol (VLP) by Z. Liu et al.

Z.Liu et al. [38] proposed variable linear shift-based authentication protocol (VLP) to support the implementation of RFID for the new EPC Gen2v2 standard, satisfy its security features of untraceability and access control, and reduce a tag’s read range. In Figure 19, the protocol is based on a lightweight encryption function called Variable Linear Feedback Shift Register (VLFSR), which is implemented at the application-specific integrated circuit (ASIC) level. In every session, mutual authentication involves different random numbers from the tag and reader combined with the new secret value SID stored in the database to provide resistance against active attacks.

**Figure 20:**
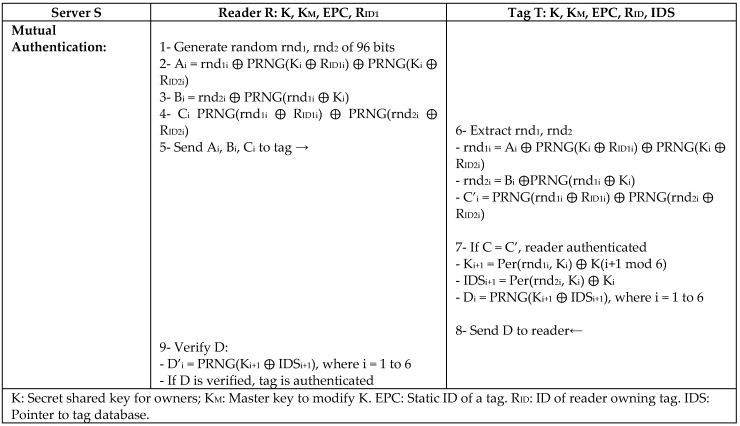
Passive Tag Ownership Authentication Protocol (OMP) Protocol by Niu.

Another protocol (OMP) is proposed by Niu et al. [39] mainly for passive tag ownership transfer using a lightweight authentication mechanism to support EPC Gen2 standard. Since the ownership transfer is based on transferring the keys, the OMP protocol aims to prove the possession of the shared secret key to a tag and reader without disclosing it using ultra-lightweight permutation operation (Per), as in Figure 20. Yet, the protocol has no mechanism to check the freshness of the message that is sent by a legitimate reader. 

**Figure 21:**
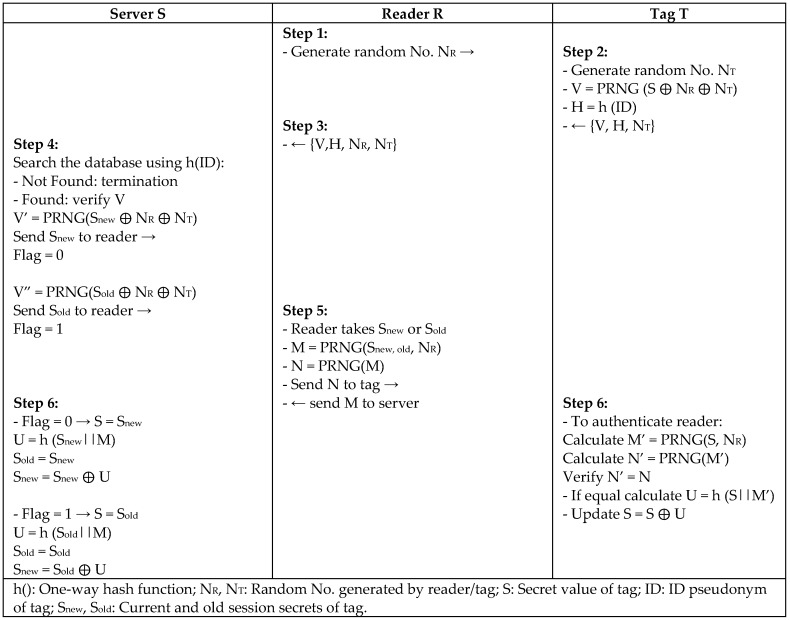
Efficient Authentication Protocol (SEAS) by Dass and Om.

Dass and Om [40] also proposed an efficient authentication protocol (SEAS) that uses lightweight operations and a pseudo-random number generator (PRNG) for a low computational cost. Their scheme is based on a secure channel between the back-end server and reader, prestored tags’ secret (SIDs) in the tags side, a one-way hash function of the tag ID in the server side, and rewritable memory with a flag indicator in the server side to update the secret values. Any change to the messages transmitted leads to terminate the communication during the verification to resist security attacks, as shown in Figure 21. 

**Figure 22:**
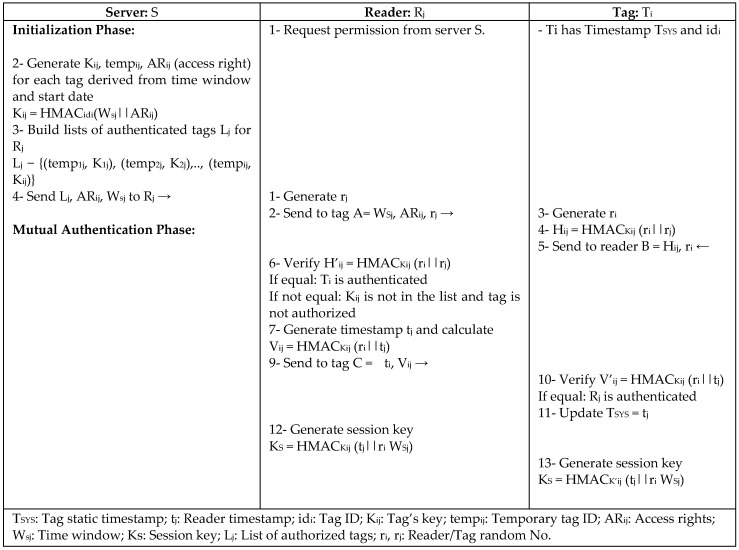
Serverless Security Authentication Protocol (SAP) by Mtita.

An alternative solution to replace the central database in the RFID system is to use a *serverless* model in which the database server does not maintain a connection with the readers and tags during the communication. Regarding this challenge, Mtita et al. [41] proposed (SAP), a serverless security protocol used for the mass authentication of RFID tags in the presence of untrusted readers. In SAP protocol, the reader and tag do not communicate with the back-end server; instead, they authenticate each other using only ephemeral of the tag’s secrets that expire within a given time, as shown in Figure 22. Verification and authentication between reader and tag are done during the authentication phase to exchange the data and generate the session key locally in both tag and reader for their next communication. The protocol has also been proved using the *CryptoVerif* tool [42], which was shown to have low computation overhead and resources.

### 4.4. Ultra-Lightweight Protocols

**Figure 23:**
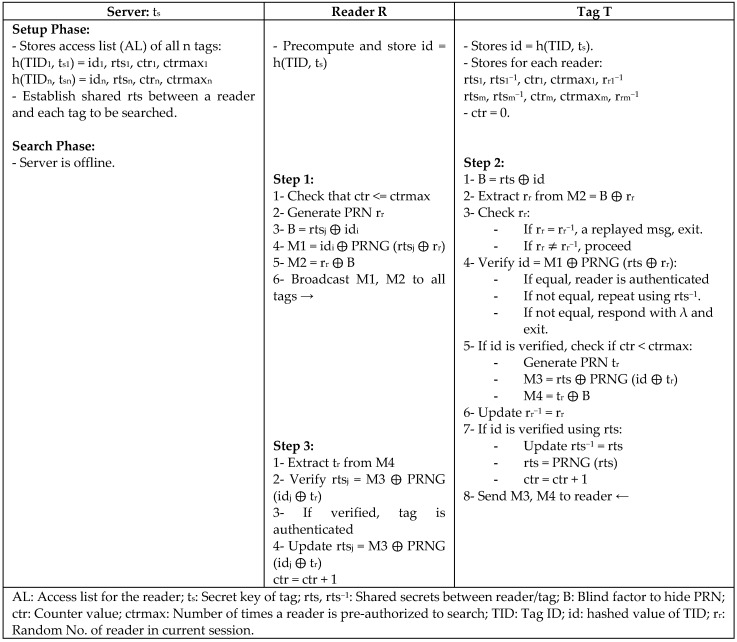
Ultra-Lightweight Serverless Authentication Protocol (STS) by Sundaresan.

As mentioned earlier in this paper, passive tags are small chips with scarce resources that can only support low-cost operations. The goal of ultra-lightweight protocols is to reduce the cost of RFID systems at a minimum and provide strong security for promising future use. In this regard, Sundaresan et al. [43] introduced an ultra-lightweight serverless protocol (STS) using only simple XOR and 128-bit PRNG operations that require less than 2000 gates, three random number generation on the tag, and two message exchanges. In Figure 23, the STS protocol mechanism is to use a blind factor to hide the pseudo-random numbers that are used in communication between readers and tags to overcome impersonation attacks. RFID tag is also able to preserve its location privacy by responding as a noise tag. Moreover, the protocol does not employ a one-way hash function nor any encryption conforming to EPC C1 G2 Standards. 

**Figure 24:**
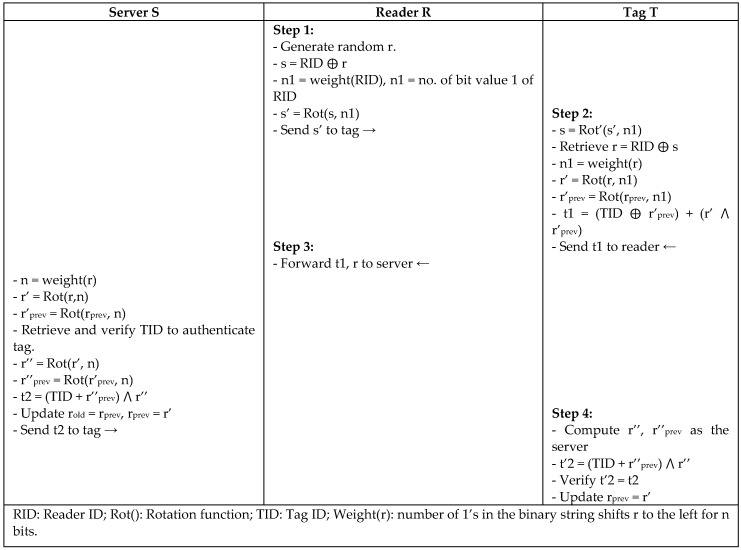
Improved Authentication Protocol (CWH+) by Aggarwal and Dass.

Aggarwal and Das [44] proposed the CHW+ protocol, which is based on a previous version introduced by Y. Chen, Wang, and Hwang (CWH) [45]. In Figure 24, the protocol CHW+ solves the problem of full disclosure attack due to the simple XOR operation that is used in the authentication message, which uses the bit rotation and shifting operation on the message before transmission to increase the protocol complexity. CWH+ protocol is resistant to replay attack, forge attack, and DoS with a very efficient computation. 

Huang and Li [46] proposed and implemented two improved protocols of RFID mutual authentication based on generating the PadGen function in the ISO 18000-6C [47] protocol to protect the memory with a 32-bit access password. The concept of their protocols is to cover up the tag’s access password (Apwd) before transmitting the data using a set of 16-bit random numbers such as RTx and RMx. One of the improved schemes, PadGen with XOR (PGX), implements XOR operation between the random number sets and the PadGen function; the other protocol, PadGen with Mod (PGM), implements a Modulo operation (MOD9) in the eight-bit half of the 16-bit random number set (RTx, RMx) to be used in the PadGen function. Both improved schemes conform to the EPC C1 G2 standard, do not require any hash function or key exchange, do not involve synchronization for hash or key values, and also show better efficiency during implementation. The security level of the MOD scheme is higher due to the low-cost implementation, but requires a higher computation cost in PadGen than XOR. 

**Figure 25:**
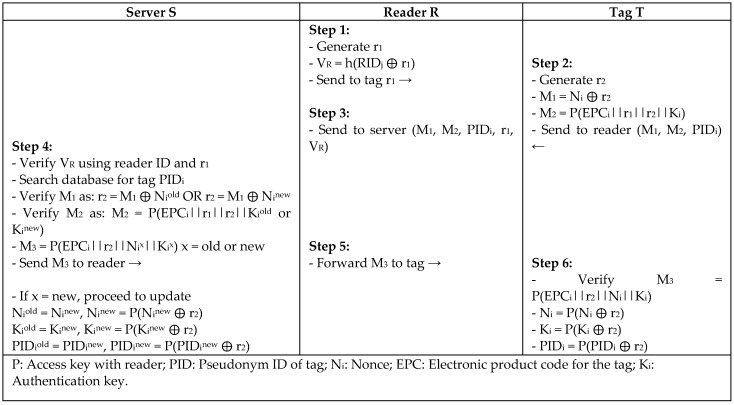
Ultra-Lightweight Reader–Tag Mutual Authentication Protocol (MACC) by Huang and Jiang.

Huang and Jiang [48] proposed an ultra-lightweight reader–tag mutual authentication protocol (MACC) based on Chien and Chen’s protocol [49] to overcome forge attacks, DoS, and forward security attacks. Although the improved scheme uses only lightweight operations such as RNG, PRNG, and XOR, it involves an exhaustive search in the database for tag pseudo-IDs in every session that leads to computational overhead, as shown in Figure 25. It also fails to resist tracking attacks. 

**Figure 26:**
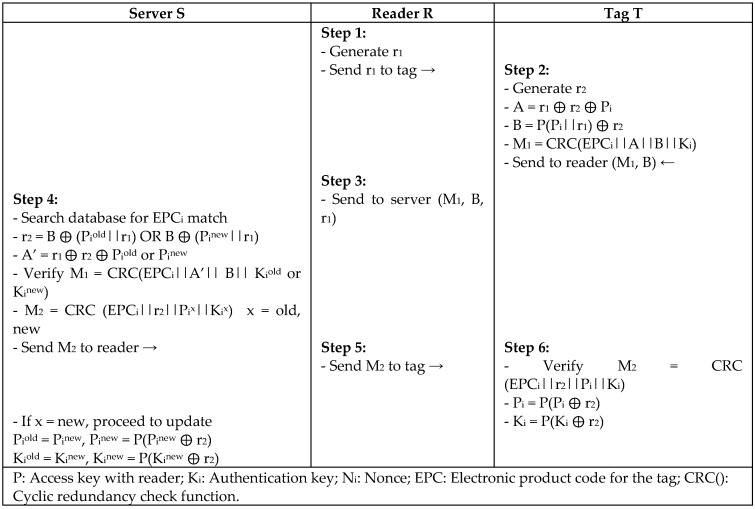
Mutual Authentication Protocol (MACD) by Huang and Jiang.

Huang and Jiang [48] proposed another mutual authentication protocol (MACD) based on Chen and Deng’s scheme [50] to overcome forge attacks, DoS, replay attacks, and mainly the tag identification time. It is shown in Figure 26 that the MACD protocol uses ultra-lightweight operations and achieves a lower communication cost between tag and reader than the other improved scheme, MACC. 

Considering the complexity of the authentication protocol, Hopper and Blum proposed the first HB protocol to identify unaided humans to computers [51]. Many authors adopted the idea of HB protocol to identify tags in RFID networks. As a matter of fact, HB family protocols are based on the hard problems of Learning Parity with Noise (LPN), which involves the calculation of inner products of binary vectors and Bernoulli noise bit generation [52]. In this regard, Lin and Song [53] proposed HBROT, which is one of the latest HB protocols that produces the key in each authentication round using the rotation function. The protocol is considered to be secure against most of the RFID attacks.

**Figure 27:**
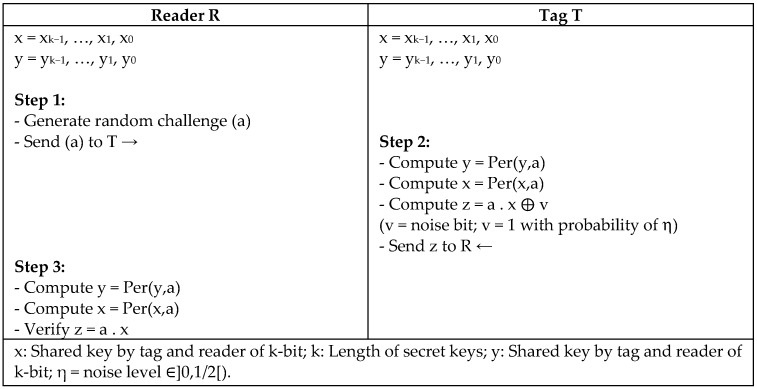
A Variant of HB Protocol Based on Permutation Function (HBPER) by Ouaskou et al.

Another improvement of the HB protocol is proposed by Juels and Weis [54] as (HB+) to overcome the weaknesses of the original HB. The HB+ protocol involves two secret keys, x and y, which are used with shared blind vectors between the reader and tag. The reader and tag verify the values that are computed to perform the mutual authentication. Later, the protocol is reported by Gilbert et al. [55] to be vulnerable to the man-in-the-middle attack (MIM). Hence, Ouaskou et al. [56] proposed a variant of HB protocol based on Permutation function (HBPER). The protocol performs a permutation of the keys x, y during each round of the protocol to update the value of the keys, as shown in Figure 27. This method secures the protocol against the MIM attack that is reported in the HB+ protocol, although both protocols HB+ and HBPER almost have the same complexity.

## 5. Analysis and Security Evaluation

In this section, we compare the different protocols in terms of computation, security requirements, and attacks resistance. Since the passive tag used in the RFID system has limited computation capabilities and resources, it is important to consider the computation and security features for the appropriate application. Table 2 demonstrates the different operations computed by the tag in each protocol, and the communication overhead based on the number of transmitted messages between tag and reader.

### 5.1. Comparison of Computation Cost

We denote T*_ENC_*, T*_DEC_*, T*_PRNG_*, T*_RNG_*, T*_SMUL_*, T*_XOR_*, T*_CH_*, T*_H_*, T*_CRC_*, T*_ROT_*, T*_SHIFT_*, T*_ITER_*, T*_BIT_*, T*_SPR_*, T*_PER_*, T*_MOD_*, T*_VLFSR_* as the computation cost for encryption, decryption, pseudo-random number generator, random number generator, scalar multiplication, XOR, cryptographic hash, one-way hash function, cyclic redundancy check, rotation, shifting, iteration, bitwise operation, spreading, permutation, modulo, variable linear shift register function, respectively. Tag overhead is classified based on the cryptographic level of operations used in the protocol: *high* for symmetric key cryptography and scalar multiplication, *medium* for one-way hash function, and *low* for other bitwise operations and random number generators. The passes are designated for the number of messages sent by a reader or a tag. 

### 5.2. Comparison of Security Threats

Protocols resistance to different RFID threats is presented in Table 3, where we denote ST1 for a replay attack, ST2 for a man-in-the-middle attack (MITM), ST3 for eavesdropping, ST4 for an impersonating attack, ST5 for traceability, ST6 for desynchronization, ST7 for denial of service (DoS), and ST8 for other types of attack. We found that most of the recently proposed protocols do not pay close enough attention to DoS, MITM, and eavesdropping attacks, while most of the protocols consider the system security against replay, impersonate, traceability, and desynchronization attacks. Certainly, protocols [6,19,20,23,34,53,56] are strongly resistant to all of the major attacks. 

### 5.3. Comparison of Security Requirements

Security requirements for an RFID system should be satisfied in order for the system to defend against the attacks mentioned in this paper. Table 4 compares the security requirements in each protocol, which includes mutual authentication (SR1), confidentiality (SR2), message integrity (SR3), privacy (SR4), forward secrecy (SR5), backward secrecy (SR6), tag anonymity (SR7), and conforming to EPC standards (SR8). We found that most of the protocols fully considered the mutual authentication, privacy, and data protection, while backward secrecy is given the least attention, and should be more considered in future work. However, Niu et al. [39] and X. Chen [23] completely satisfied all of the security requirements in their protocol. 

## 6. Conclusions

Recent RFID authentication protocols are proposed to develop an efficient and secure RFID system. This survey is conducted to review and compare different RFID authentication protocols of low-cost passive tags for better utilization in the appropriate application. We demonstrate in this study the security requirements of an RFID system that must be satisfied, so the system could be able to defend major attacks such as replay, man-in-the-middle, impersonation, desynchronization, DoS, and more. We further identify the category levels of the protocols based on the operation complexity on the tag side, and compare the protocols based on the tag computation cost. Since the RFID passive tag has limited resources to compute complex operations, the heavyweight and simple-weight protocols are not feasible for practical implementation. However, lightweight and ultra-lightweight protocols use only simple operations within the tag computation limits, and show the lowest tag overhead level. Lightweight and ultra-lightweight protocols are considered the most suitable for the current applications. Another vital aspect when considering the appropriate RFID protocol is the security resistance to the attacks. We examined the security threats in each protocol presented in the review. We found out that Chang et al. [6], Farash [19], Zhang and Qi [20], X. Chen et al. [23], Liu et al. [34], Lin and Song [53], and Ouaskou et al. [56] protocols successfully resist all of the major attacks. Although the other protocols could not resist all of the attacks, they could perform better than the fully secure protocols in term of computation cost; examples include the protocols presented in Farash [19], Zhang and Qi [20], X. Chen et al. [23], and Liu et al. [34], which have high computation overhead on the tag side. We encourage researchers to pay attention to the forward and backward security, since most protocols do not reflect on these two types of attacks. Finally, maintaining the basic security requirements for an RFID system is required to achieve protection against the mentioned attacks in this article. Our assessment is that only the protocols of Niu et al. [39] and X. Chen et al. [23] satisfy all of the security requirements to maintain the system in a stable and available state. Even though this review shows security variation among the reviewed protocols, each one could still be a preference over others, depending on the requirements of the application in hand. 

## Figures and Tables

**Table 1:** Classification of RFID Tags [7,8].

	Passive Tags	Semi-Passive Tags	Active Tags
**Power**	Surrounding signal	Internal chip battery	Integrated battery
**Storage**	Read memory	Reade/write memory	Reade/write memory
**Distance**	5 m	100 m	1000 m
**Application**	Identification	Real-time tracking	Environmental and logistic
**Cost**	Low	High	High
**Size**	Small	Large	Large
**Lifespan**	Unlimited	10 years	10 years
**Tag Signal**	Low	High	High
**Required Signal**	High	Low	Low

**Table 2:** Comparison of the Computation Cost on Tag.

Protocol	Operations	Tag Passes	Reader Passes	Tag Overhead
SB-A [11]	1 T*_ENC_* + 2 T*_DEC_* + 2 T*_PRNG_*	2	3	High
SB-B [11]	2 T*_ENC_* + 2 T*_DEC_* + 2 T*_PRNG_*	2	3	High
EMA [15]	2 T*_SMUL_* + 2 T*_CH_*	2	1	High
ECU [13]	2 T*_SMUL_* + 2 T*_CH_*	1	1	High
SPA [14]	4 T*_SMUL_* + 1 T*_CH_*	1	1	High
PII [16]	4 T*_SMUL_* + 3 T*_CH_*	1	1	High
RUND [17]	2 T*_H_* OR 1 T*_ENC_* + 1 T*_PRNG_*	1	2	High
IECC [19]	2 T*_SMUL_* + 2 T*_H_*	1	2	High
EECC [20]	2 T*_SMUL_* + 2 T*_H_*	1	2	High
RBAC [21]	2 T*_ENC_* + 2 T*_DEC_* + 1 T*_PRNG_*	2	2	High
DRAP [33]	1 T*_ENC_* + 3 T*_XOR_* + 3 T*_H_* + 1 T*_RNG_* + 2 T*_PRNG_*	1	2	High
NRS [22]	10 T*_XOR_* + 3 T*_H_*	4	5	Medium
NRS+ [10]	10 T*_XOR_* + 6 T*_H_*	4	5	Medium
NRS++ [23]	8 T*_XOR_* + 4 T*_H_*	1	2	Medium
ACSP [24]	3 T*_XOR_* + 7 T*_H_* + 4 T*_CRC_*	1	4	Medium
ACSP+ [25]	4 T*_XOR_* + 8 T*_H_*	2	4	Medium
ACSP++ [23]	6 T*_XOR_* + 8 T*_H_*	1	2	Medium
MASS [31]	4 T*_XOR_* + 2 T*_H_* + 1 T*_RNG_*	1	2	Medium
EP-UAP [32]	2 T*_H_* + 1 T*_RNG_*	1	2	Medium
GUPA [34]	2 T*_H_* + 3 T*_PRNG_* + 19 T*_BIT_*	3	3	Medium
HBA [37]	6 T*_XOR_* + 2 T*_H_* + 1 T*_RNG_* + 4 T*_MOD_*	1	2	Medium
VLP [38]	2 T*_XOR_* + 2 T*_RNG_* + 3 T*_BIT_* + 2 T*_VLFSR_*	1	2	Medium
SEAS [40]	1 T*_XOR_* + 2 T*_H_* + 1 T*_RNG_* + 3 T*_PRNG_* + 1 T*_BIT_*	1	2	Medium
SAP [41]	2 T*_H_* + 2 T*_RNG_*	1	2	Medium
LAP [26]	2 T*_XOR_* + 1 T*_RNG_* + 2 T*_PRNG_* + 1 T*_ROT_* + 1 T*_SHIFT_*	2	2	Low
Flyweight [29]	5 T*_PRNG_*	3	3	Low
FTest [35]	1 T*_XOR_* + 3 T*_CRC_*	3	2	Low
ACS [36]	3 T*_XOR_* + 2 T*_ITER_* + 1 T*_SPR_*	1	2	Low
HBA+ [6]	7 T*_XOR_* + 1 T*_RNG_* + 5 T*_PRNG_*	1	2	Low
OMP [39]	12 T*_XOR_* + 6 T*_PRNG_* + 2 T*_PER_*	1	1	Low
STS [43]	7 T*_XOR_* + 3 T*_PRNG_*	1	1	Low
CWH+ [44]	2 T*_XOR_* + 5 T*_ROT_* + 1 T*_SHIFT_* + T*_BIT_*	1	1	Low
PGX [46]	8 T*_XOR_* + 2 T*_RNG_*	2	2	Low
PGM [46]	4 T*_XOR_* + 2 T*_RNG_* + 32 T*_MOD_*	2	2	Low
MACC [48]	6 T*_XOR_* + 5 T*_PRNG_*	1	2	Low
MACD [48]	5 T*_XOR_* + 3 T*_PRNG_* + 1 T*_CRC_*	1	2	Low
HBROT [53]	1 T*_RNG_* + 2 T*_ROT_* + 1 T*_XOR_* + 1 T*_BIT_*	1	1	Low
HBPER [56]	1 T*_RNG_* + 2 T*_PER_* + 1 T*_XOR_* + 1 T*_BIT_*	1	1	Low

T*_ENC_*: encryption, T*_DEC_*: decryption, T*_PRNG_*: pseudo-random number generator, T*_RNG_*: random number generator, T*_SMUL_*: scalar multiplication, T*_XOR_*: XOR, T*_CH_*: cryptographic hash, T*_H_*: one-way hash function, T*_CRC_*: cyclic redundancy check, T*_ROT_*: rotation, T*_SHIFT_*: shifting, T*_ITER_*: iteration, T*_BIT_*: bitwise operation, T*_SPR_*: spreading, T*_PER_*: permutation, T*_MOD_*: modulo, T*_VLFSR_*: variable linear shift register function.

**Table 3:** Comparison of Various Security Threats Resistance.

	ST1	ST2	ST3	ST4	ST5	ST6	ST7	ST8
SB-A [11]	Y	Y	Y	Y	Y	Y	*	Cloning
SB-B [11]	Y	Y	Y	Y	Y	Y	*	Cloning
ECU [13]	Y	Y	*	Y	Y	*	*	*
SPA [14]	N	*	*	N	Y	*	*	*
EMA [15]	Y	*	*	N	N	*	*	*
PII [16]	Y	*	*	Y	Y	*	*	*
RUND [17]	Y	*	*	Y	Y	Y	Y	*
IECC [19]	Y	Y	Y	Y	Y	Y	Y	Cloning
EECC [20]	Y	Y	Y	Y	Y	Y	Y	Spoofing
RBAC [21]	Y	*	*	Y	Y	*	Y	*
NRS [22]	N	Y	N	N	N	N	N	*
NRS+ [10]	N	Y	Y	N	N	N	N	*
NRS++ [23]	Y	Y	Y	Y	Y	Y	Y	*
ACSP [24]	N	N	N	N	N	N	N	Counting
ACSP+ [25]	N	*	*	N	Y	Y	N	Counting
ACSP++ [23]	Y	Y	Y	Y	Y	Y	Y	Counting
LAP [26]	Y	*	*	N	N	N	Y	*
Flyweight [29]	Y	Y	Y	Y	Y	Y	*	*
MASS [31]	N	N	N	N	Y	N	*	*
EP-UAP [32]	N	Y	Y	N	Y	*	*	*
DRAP [33]	Y	*	*	Y	Y	Y	Y	Y
GUPA [34]	Y	Y	Y	Y	Y	Y	Y	DoP
FTest [35]	Y	Y	Y	Y	Y	*	*	Counterfeit + Collision
ACS [36]	Y	Y	Y	Y	Y	*	*	Counterfeit + Collision
HBA [37]	N	Y	Y	Y	Y	Y	N	Brute + Counterfeit
HBA+ [6]	Y	Y	Y	Y	Y	Y	Y	Brute for
VLP [38]	Y	Y	Y	*	Y	Y	*	*
OMP [39]	N	*	*	Y	Y	Y	Y	*
SEAS [40]	Y	Y	*	Y	Y	Y	Y	*
SAP [41]	Y	*	Y	Y	Y	*	*	*
STS [43]	Y	*	*	Y	Y	Y	Y	*
CWH+ [44]	Y	*	Y	Y	*	Y	*	Disclosure
PGX [46]	Y	Y	Y	Y	N	Y	*	Cloning
PGM [46]	Y	Y	Y	Y	N	Y	*	Cloning
MACC [48]	Y	Y	*	Y	N	Y	Y	*
MACD [48]	Y	Y	*	Y	Y	Y	Y	*
HBROT [53]	Y	Y	Y	Y	Y	Y	Y	*
HBPER [56]	Y	Y	Y	Y	Y	Y	Y	*

ST1: replay attack, ST2: man-in-the-middle, ST3: eavesdropping, ST4: impersonate attack, ST5: traceability, ST6: desynchronization, ST7: DoS, ST8: other types of attack, Y: satisfied, N: not satisfied. *: not applicable.

**Table 4:** Comparison of the Security Requirements.

	SR1	SR2	SR3	SR4	SR5	SR6	SR7	SR8
SB-A [11]	Y	Y	Y	Y	Y	*	Y	N
SB-B [11]	Y	Y	Y	Y	Y	*	Y	N
ECU [13]	N	Y	Y	Y	Y	*	Y	N
SPA [14]	*	*	*	*	N	*	*	*
EMA [15]	*	*	*	*	N	*	*	*
PII [16]	*	*	*	*	N	*	*	*
RUND [17]	Y	Y	Y	Y	Y	*	Y	N
IECC [19]	Y	Y	Y	Y	Y	Y	Y	N
EECC [20]	Y	Y	Y	Y	Y	Y	Y	N
RBAC [21]	Y	Y	Y	Y	*	*	Y	N
NRS [22]	N	Y	N	N	N	N	N	Y
NRS+ [10]	N	Y	Y	N	N	N	N	Y
NRS++ [23]	Y	Y	Y	Y	Y	Y	Y	Y
ACSP [24]	Y	N	N	N	N	N	N	Y
ACSP+ [25]	Y	Y	Y	*	N	Y	*	Y
ACSP++ [23]	Y	Y	Y	Y	Y	Y	*	Y
LAP [26]	Y	Y	Y	N	Y	*	N	Y
Flyweight [29]	Y	Y	Y	Y	Y	Y	Y	Y
MASS [31]	Y	Y	N	*	Y	*	*	Y
EP-UAP [32]	N	Y	Y	Y	*	*	Y	Y
DRAP [33]	Y	*	*	Y	*	*	Y	Y
GUPA [34]	Y	Y	Y	Y	Y	*	Y	Y
FTest [35]	N	Y	Y	Y	Y	*	Y	Y
ACS [36]	Y	*	*	Y	*	*	Y	Y
HBA [37]	Y	Y	Y	Y	Y	*	Y	Y
HBA+ [6]	Y	Y	Y	Y	Y	*	Y	Y
VLP [38]	Y	Y	Y	Y	Y	*	Y	Y
OMP [39]	Y	Y	Y	Y	Y	Y	Y	Y
SEAS [40]	Y	Y	Y	Y	Y	*	Y	Y
SAP [41]	Y	Y	Y	*	*	*	*	Y
STS [43]	Y	Y	Y	Y	Y	*	Y	Y
CWH+ [44]	Y	Y	Y	*	Y	*	*	Y
PGX [46]	Y	*	*	N	*	*	N	Y
PGM [46]	Y	*	*	N	*	*	N	Y
MACC [48]	Y	Y	Y	N	Y	*	N	Y
MACD [48]	Y	Y	Y	Y	Y	*	Y	Y
HBROT [53]	Y	Y	Y	Y	Y	*	*	Y
HBPER [56]	Y	Y	Y	Y	Y	*	*	Y

SR1: mutual authentication, SR2: confidentiality, SR3: message integrity, SR4: privacy, SR5: forward secrecy, SR6: backward secrecy, SR7: tag anonymity, SR8: conforming to EPC standard, Y: satisfied, N: not satisfied, *: not applicable.
